# Burr Segmentation Method for Oil Injection Holes of High-Speed Train Wheelsets Based on Improved PointNet++

**DOI:** 10.3390/s26216735

**Published:** 2026-10-24

**Authors:** Shuang Zhang, Jiao Yu, Hongli Lv, Jinwei Du, Qingping Zhang, Mengyu Zhou

**Affiliations:** 1 School of Mechanical and Electrical Engineering, Changchun Institute of Technology, Changchun 130012, China; 2 School of Energy and Power Engineering, Changchun Institute of Technology, Changchun 130012, China; 20241031250@stu.ccit.edu.cn (J.Y.);; 3 School of Mechatronical Engineering, Changchun University of Science and Technology, Changchun 130012, China

**Keywords:** high-speed train wheelsets, oil injection-hole burrs, point cloud segmentation, PointNet++

## Abstract

Accurate segmentation of burrs in the oil injection holes of high-speed train wheelsets is essential for automated quality inspection. To address the small size, irregular morphology, and indistinct local boundary features of these burrs, we propose an improved PointNet++-based point cloud segmentation method for oil injection-hole burrs. First, we established an experimental platform for acquiring point clouds of oil injection-hole burrs and used a line laser sensor to acquire three-dimensional point clouds of the oil injection-hole region. We then preprocessed the acquired point clouds to construct a dataset containing burrs with diverse morphologies. Subsequently, to address the indistinct local structural features of burrs, we incorporated a Kernel Point Convolution (KPConv) module into the shallow feature extraction stage of PointNet++ to strengthen the network’s ability to extract local geometric features of burrs. On this basis, we designed a Kernel-Guided Geometric Point Attention (KGPA) module. By jointly incorporating point features, relative coordinates, local density, and distances to kernel points, KGPA assigns weights to neighboring features, thereby enabling the network to focus more on burr boundaries and small protruding regions. Experimental results show that the proposed network achieved an mIoU of 94.25%, an OA of 98.38%, a recall of 98.65%, and a precision of 98.50%, representing improvements of 2.28, 1.62, 2.25, and 2.34 percentage points, respectively, over PointNet++. The results demonstrate that the proposed method can accurately segment oil injection-hole burrs from point clouds, providing technical support for subsequent burr localization, size evaluation, and determination of removal regions.

## 1. Introduction

High-speed train wheelsets are critical load-bearing components in train operating systems, and their manufacturing quality directly affects operational safety and service life [1]. As a key oil passage during wheelset press-fitting and removal, the oil injection hole delivers high-pressure oil and facilitates the formation of an oil film at the mating interface between the wheel hub bore and the axle wheel seat [2]. During drilling, however, tool cutting and material deformation can generate local burrs around the hole opening and along the hole wall [3]. Residual burrs degrade the surface quality of the oil injection hole and may adversely affect subsequent assembly and operation. Therefore, manufacturers must accurately detect and promptly remove these burrs. Although vision-based methods have been explored for burr inspection [4], the small size, irregular three-dimensional morphology, and complex spatial distribution of oil injection-hole burrs still pose challenges to reliable detection, particularly around the hole opening and along the hole wall. As wheelset manufacturing becomes increasingly automated and intelligent, this trend demands higher accuracy and efficiency in component defect inspection. Therefore, investigating a non-contact and nondestructive inspection technique capable of identifying minute burr defects in oil injection holes is important for improving inspection accuracy and automation and for providing a reliable basis for subsequent burr size measurement and automated removal.

In recent years, deep learning-based point cloud segmentation methods have been increasingly applied to industrial defect inspection [5]. According to how they represent point clouds, existing methods fall into three categories: voxel-based [6], projection-based [7], and point-based methods [8]. Voxel-based and projection-based methods convert point clouds into regular representations, which may result in the loss of local three-dimensional geometric information. By contrast, point-based methods directly process unordered point clouds and better preserve spatial locations and local geometric information, making them more suitable for segmenting oil injection-hole burrs. PointNet++ progressively extracts local geometric features from raw point clouds through hierarchical sampling, local neighborhood grouping, and multiscale feature aggregation. Its set abstraction modules also offer good modularity, facilitating modifications tailored to the small scale, irregular morphology, and inconspicuous boundary features of oil injection-hole burrs. We therefore selected PointNet++ as the baseline network. However, PointNet++ mainly uses shared multilayer perceptrons and max pooling to extract neighborhood features but does not adequately exploit the positional relationships among neighboring points. During hierarchical sampling, the local features of small burrs may gradually weaken, thereby increasing the likelihood of missegmentation. Moreover, PointNet++ cannot readily assign different levels of importance to neighboring points during feature extraction and therefore pays insufficient attention to critical burr features. It is thus necessary to strengthen the network’s ability to represent local geometric structures and increase its focus on burrs and their boundary regions.

To address these problems, we propose a PointNet++-based method for segmenting burrs in the oil injection holes of high-speed train wheelsets. First, we employ a KPConv module in the shallow feature extraction stage of PointNet++ to strengthen the network’s extraction of local geometric features of burrs. Second, we design a KGPA module to further enhance the network’s representation of critical burr regions. The main contributions of this study are as follows:

(1) We have developed a high-precision 3D point cloud data acquisition and processing process for detecting burrs in the oil injection holes of high-speed train wheelsets, including steps such as line laser point cloud acquisition, point cloud downsampling, and point-level semantic annotation. This provides a reliable data foundation for the training and performance evaluation of subsequent oil injection hole burr point cloud segmentation models.

(2) To address the small local protrusions and complex boundary morphology of oil injection-hole burrs, we introduced KPConv after the first set abstraction layer (SA1) of PointNet++. KPConv aggregates features according to the spatial relationships between virtual kernel points and neighboring points, thereby strengthening the network’s ability to extract local geometric features of burrs.

(3) We designed a KGPA module that uses virtual kernel points as local geometric references and combines point features, relative coordinates, local density, and distances to kernel points to generate attention weights. Through kernel-wise feature aggregation, the module strengthens the representation of burr edges and small protruding regions, thereby improving the segmentation performance for oil injection-hole burr point clouds.

## 2. Related Work

### 2.1. Deep Learning-Based Point Cloud Segmentation Methods

In recent years, deep learning-based point cloud segmentation methods have been widely applied to three-dimensional scene understanding and industrial inspection. Compared with conventional methods that rely on manually designed geometric features, deep learning methods can directly learn spatial distributions and local structural features from unordered point clouds, reducing their dependence on handcrafted features. Qi et al. [9] proposed PointNet++, which progressively extracts local geometric features through hierarchical sampling, local neighborhood grouping, and multiscale feature aggregation. This architecture provides a fundamental framework for point cloud segmentation. Subsequent studies have further improved the segmentation performance of PointNet++ by modifying its local feature extraction and feature aggregation strategies. For example, Wang et al. [10] introduced a coordinate attention module into the PointNet++ backbone for semantic segmentation of three-dimensional point clouds from transmission corridors, achieving an overall accuracy of 93.7% and a mean intersection over union (mIoU) of 67.4%. Zhang et al. [11] incorporated a self-attention mechanism into the set abstraction modules of PointNet++ and integrated a local feature extraction module from PointNet-Transformer to segment geometric features such as holes, arcs, and surfaces in mechanical components, achieving a segmentation accuracy of 86.6% and an mIoU of 0.84. Zhong et al. [12] introduced a cross-attention mechanism into PointNet++ to segment point clouds of planar and curved weld regions, achieving a segmentation accuracy of 98.87% and an mIoU of 90.71%. These studies demonstrate that PointNet++ can effectively extract local point cloud features and offers good architectural extensibility. By incorporating different feature enhancement modules, researchers can adapt PointNet++ to various point cloud segmentation tasks.

### 2.2. Point Cloud Segmentation Methods for Minute Defects

Research on point cloud segmentation for minute industrial defects remains relatively limited. Because such defects are typically small, contain few points, and exhibit subtle local geometric variations, existing studies have mainly enhanced segmentation performance by strengthening local geometric features, introducing attention mechanisms, and refining feature modeling strategies. For example, Zhang et al. [13] proposed a weakly supervised network, WS-CCNet, for segmenting small protrusion defects on cast workpiece surfaces. The network uses a central-channel dual-attention mechanism to enhance defect-related features and employs geometry-aware positional encoding to integrate geometric information such as position, orientation, and curvature, thereby improving the recognition of minute defects. Pan et al. [14] proposed a Local Graph Attention Semantic Segmentation network (LGASS) for three-dimensional point cloud segmentation of surface defects on sealing nails for automotive power batteries. LGASS constructs local graph attention using differences between the normal vectors of neighboring points and employs spatial attention graph pooling to preserve critical geometric points during downsampling, thereby strengthening the extraction of subtle geometric surface anomalies. Their method achieved an OA of 99.47% and an mIoU of 79.23%. Guo et al. [15] constructed the CPS3D-Seg point cloud defect dataset and proposed a causal inference-based network, CINet, to address the difficulty of accurately segmenting minute surface defects on integrated-circuit ceramic packaging substrates. CINet strengthens point cloud feature representation through a structure optimization module and a quality assessment module, thereby improving minute-defect segmentation. Although these methods improve the segmentation of minute defects through geometric feature enhancement and attention mechanisms, they mainly target protrusions, depressions, and other defects on external workpiece surfaces. For oil injection-hole burrs distributed around the hole opening and along the hole wall, the extraction of subtle local geometric features and boundary information still requires improvement.

### 2.3. KPConv-Based Local Feature Extraction for Point Clouds

Local geometric features in point clouds reflect the spatial distributions, surface variations, and boundary changes among neighboring points and therefore provide important information for distinguishing different regions. Thomas et al. [16] proposed Kernel Point Convolution (KPConv), which places multiple virtual kernel points in three-dimensional space and computes influence weights according to the distances between neighboring points and kernel points. KPConv then aggregates neighboring point features using these weights, enabling it to extract local geometric features from the spatial relationships among points. Bi et al. [17] employed KPConv in the point feature extraction branch of a point cloud semantic segmentation network. KPConv aggregates features using the spatial relationships between neighboring points and virtual kernel points to extract fine-grained point features from raw point clouds. The network then fuses these features with multiscale voxel features to strengthen its representation of local spatial structures. Oil injection-hole burrs are small and directly connected to the normal hole wall. Their differences from the hole wall mainly appear as subtle geometric features, including local protrusions, surface variations, and boundary changes. Because KPConv extracts local geometric information from the spatial relationships between neighboring points and kernel points, it is well suited to extracting the features of oil injection-hole burrs. We therefore introduce KPConv into the shallow feature extraction stage of PointNet++ to strengthen the extraction of subtle burr protrusions and boundary variations.

### 2.4. Geometry-Based Attention for Point Cloud Feature Enhancement

Different neighboring points contribute unequally to the feature representation of a center point. Attention mechanisms assign different aggregation weights to neighboring points according to their features and spatial relationships, thereby highlighting local regions that contain important information. To incorporate geometric structures into attention weights, previous studies have introduced relative positions, distances, and local geometric features into attention calculations. For example, Shi et al. [18] proposed the Position-Feature Attention Network (PFA-Net), which combines positional encoding with Point Transformer to incorporate both spatial positions and point features during feature aggregation. Liang et al. [19] proposed the Multilevel Geometric Feature Embedding Transformer network (MGFE-T), which embeds local geometric features, including linearity, planarity, sphericity, and curvature, into a Transformer to strengthen the representation of local point cloud structures. Wu et al. [20] proposed the Geometry-Enhanced Network (GENet), which introduces spatial geometric priors into attention calculations. GENet calculates Euclidean distances between points from their spatial positions and maps these distances to geometric attention weights through a Gaussian kernel, thereby strengthening geometric information during feature aggregation. These studies demonstrate that incorporating geometric information into attention calculations can improve the representation of local structures. However, existing methods mainly compute attention weights using relative positions, interpoint distances, or local shape features and rarely exploit multiple local geometric references jointly with local density information. To address interference from normal hole-wall points in the representation of critical burr regions, we design a KGPA module that uses multiple virtual kernel points as local geometric references. The module integrates distances to kernel points, relative coordinates, and local density information and calculates attention weights for neighboring points through kernel-wise feature aggregation. In this way, it strengthens the representation of burr edges, small protruding regions, and local geometric variations.

## 3. Materials and Methods

### 3.1. Data Acquisition

**Figure 1:**
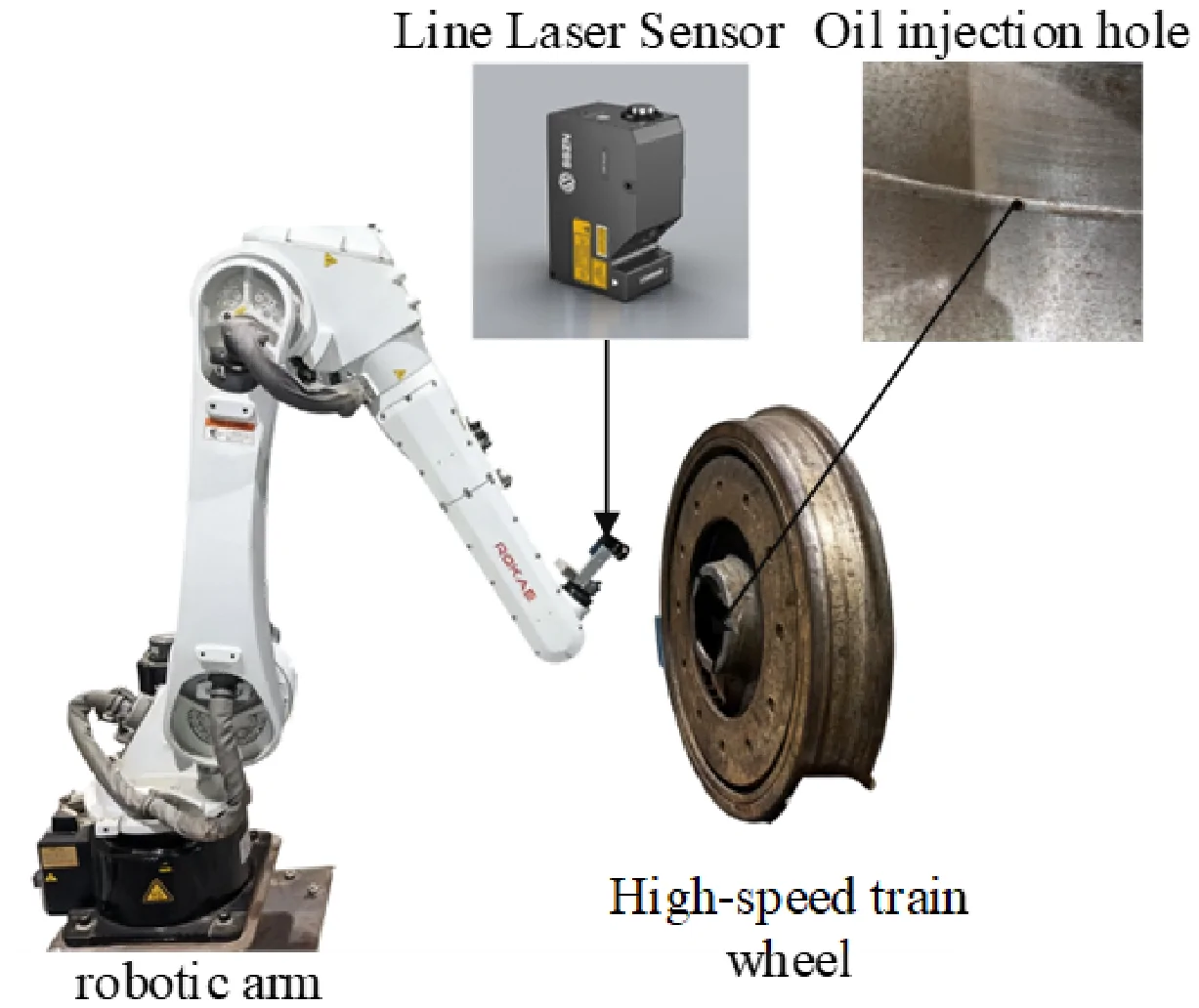
Experimental Data Acquisition Platform.

To acquire high-precision three-dimensional data from the oil injection-hole region of high-speed train wheelsets, we constructed an experimental platform for detecting wheelset oil injection-hole burrs, as shown in Figure 1. The platform mainly consisted of a robot control cabinet, high-speed train wheelsets, an XB20s-r1813-040 six-degree-of-freedom robot (ROKAE (Beijing) Robotics Co., Ltd., Beijing, China), an SR7020 line laser sensor (Shenzhen SinceVision Technology Co., Ltd., Shenzhen, China), and a host computer.

During data acquisition, we secured the wheelset specimen on a dedicated support device to maintain a stable pose. The industrial robot carried the line laser sensor along a predefined acquisition path to the oil injection-hole region. We recorded the real-time pose of the robot end effector and the acquisition pose corresponding to the center of the oil injection hole to ensure consistency across samples. Table 1 lists the main technical specifications of the line laser sensor.

### 3.2. Point Cloud Preprocessing

#### 3.2.1. Point Cloud Downsampling

Raw point clouds acquired using a line laser sensor usually contain substantial redundancy. Directly feeding these point clouds into a deep learning network would increase the computational burden. Therefore, we downsampled the raw point clouds before model training to reduce data redundancy and improve data quality.

**Figure 2:**
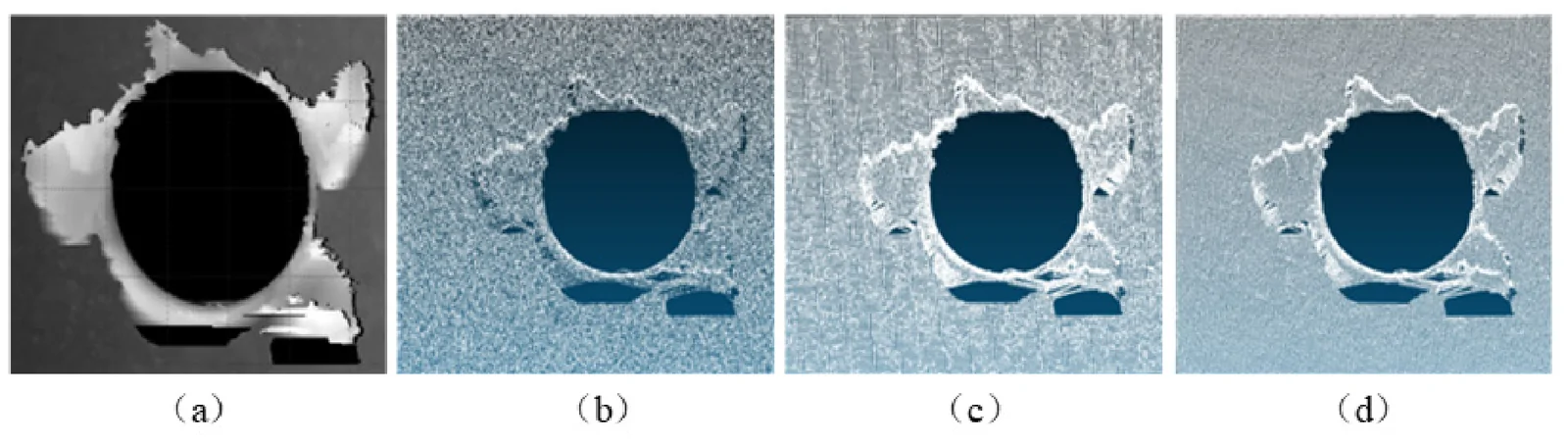
Downsampling results of the burr point cloud: (**a**) original burr point cloud; (**b**) random downsampling; (**c**) Octree-based downsampling; (**d**) voxel grid filtering-based downsampling.

Common point cloud downsampling methods include random downsampling [21], octree-based downsampling [22], and voxel grid downsampling [23]. Random downsampling is easy to implement, but its stochastic nature may cause the loss of burr boundary points. Octree-based downsampling simplifies point clouds according to a spatial hierarchy, but the resulting point clouds are strongly affected by the selected spatial partitioning level. Voxel grid downsampling divides the point cloud into regular voxels and produces a more uniform point distribution. It reduces the number of points while largely preserving their original spatial distribution. Therefore, we used voxel grid downsampling to preprocess the raw point clouds. Figure 2 compares the results obtained using different downsampling methods.

Because the voxel size directly affects the preservation of burr boundaries and local protrusions, its selection must account for the geometric scale of the burrs, preservation of burr morphology, and computational efficiency. To determine an appropriate voxel size, we statistically analyzed the depths of the acquired oil injection-hole burrs, as summarized in Table 2. The burr depths ranged from 0.410 to 1.099 mm, with a mean of 0.816 mm and a standard deviation of 0.117 mm. To prevent downsampling from weakening burr boundaries and local protrusions, the voxel size should be substantially smaller than the typical geometric scale of the burrs. Considering the burr-depth distribution, point cloud redundancy, and network training efficiency, we selected a voxel size of 0.05 mm. This value corresponds to approximately 6.1% of the mean burr depth and effectively reduces the number of points while preserving local geometric details, thereby providing standardized input for subsequent training of the point cloud segmentation model.

#### 3.2.2. Point Cloud Annotation

**Figure 3:**
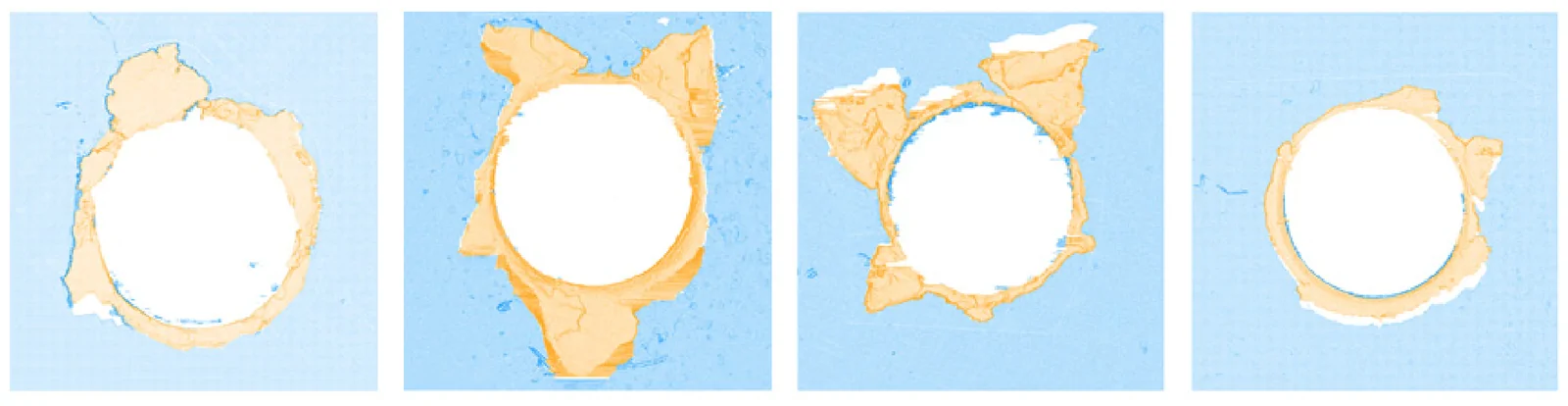
Marking Results of Burr Point Clouds in Oil Injection Holes. Yellow indicates burr points, and blue indicates hole-wall points.

To obtain ground-truth labels for the oil injection-hole burr point cloud segmentation task, we manually annotated the acquired point clouds using CloudCompare, as shown in Figure 3. Two researchers with experience in point cloud processing performed the annotation. Before annotation, they established consistent criteria for distinguishing burrs from normal hole-wall regions to improve annotation reliability.

The annotation workflow comprised the following steps:

1. We imported the preprocessed point clouds into CloudCompare and used its integrated segmentation tools to precisely separate and semantically annotate the burr regions.

2. We recombined the segmented and annotated burr regions with the normal hole-wall regions to form complete oil injection-hole burr point cloud samples. We then exported files containing XYZ coordinates, RGB color values, and semantic class labels for subsequent network training and testing. Yellow denotes burrs, whereas blue denotes normal hole-wall regions.

3. We completed point-level semantic annotation of the original oil injection-hole burr point cloud samples and saved them in PLY format. The resulting class-labeled point clouds provided data for subsequent data augmentation and network training.

### 3.3. Improved PointNet++ Network Architecture

PointNet++ is a well-established network for processing unordered point clouds. It processes point clouds hierarchically using set abstraction (SA) layers and feature propagation (FP) layers. The SA layers extract local features through neighborhood grouping, shared multilayer perceptrons (MLPs), and max-pooling operations, whereas the FP layers restore point-level features through feature interpolation and fusion. However, this feature extraction approach still has limitations when applied to the segmentation of burrs in the oil injection holes of high-speed train wheelsets. The original PointNet++ architecture does not adequately characterize local spatial distributions and boundary transitions, leading to missed burr regions and incomplete boundaries.

**Figure 4:**
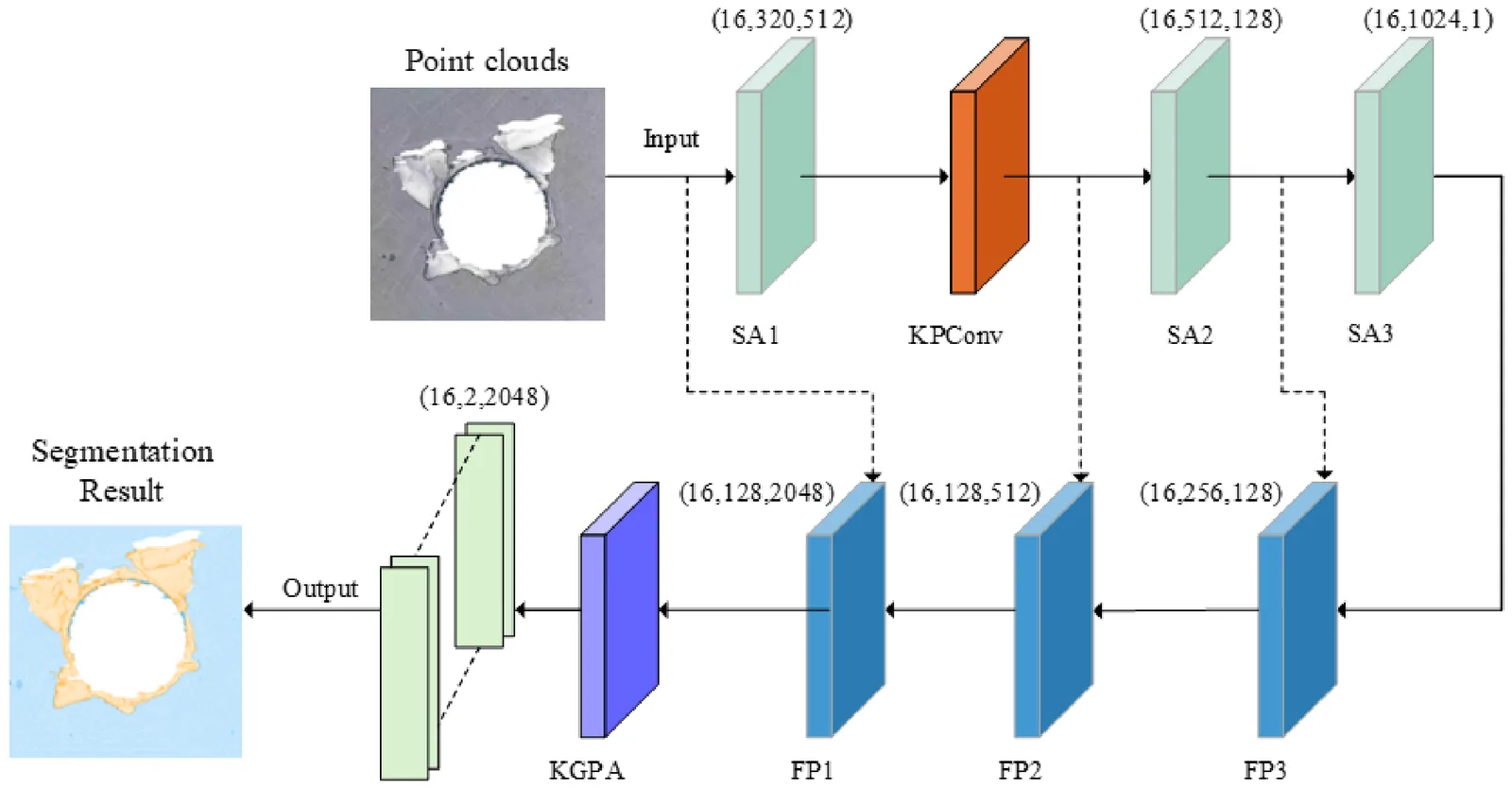
Overall architecture of the improved PointNet++ network. The numbers in parentheses indicate tensor dimensions (batch size, feature channels, and number of points). Different colors distinguish the network modules, and dashed lines indicate skip connections between the feature extraction and feature propagation stages.

To effectively extract features of burr regions and improve semantic segmentation accuracy, we developed a kernel-guided geometry-aware point cloud segmentation network based on PointNet++. We introduced a KPConv module after the first set abstraction layer (SA1) to model the spatial relationships among neighboring points using kernel points, thereby strengthening the network’s ability to represent local spatial structures. We then placed a KGPA module after FP1, the final feature propagation layer. The KGPA module calculates attention weights for neighboring points using relative coordinates, kernel-point distances, and local geometric information. This design adaptively enhances critical point features associated with burr regions while suppressing interference from background points on the hole wall. Finally, a shared MLP produces pointwise classification results as an *N* × *C* matrix, where N denotes the number of points and C denotes the number of segmentation classes. The network thereby performs semantic segmentation of oil injection-hole burr point clouds. Figure 4 illustrates the overall network architecture.

#### 3.3.1. KPConv Module

**Figure 5:**
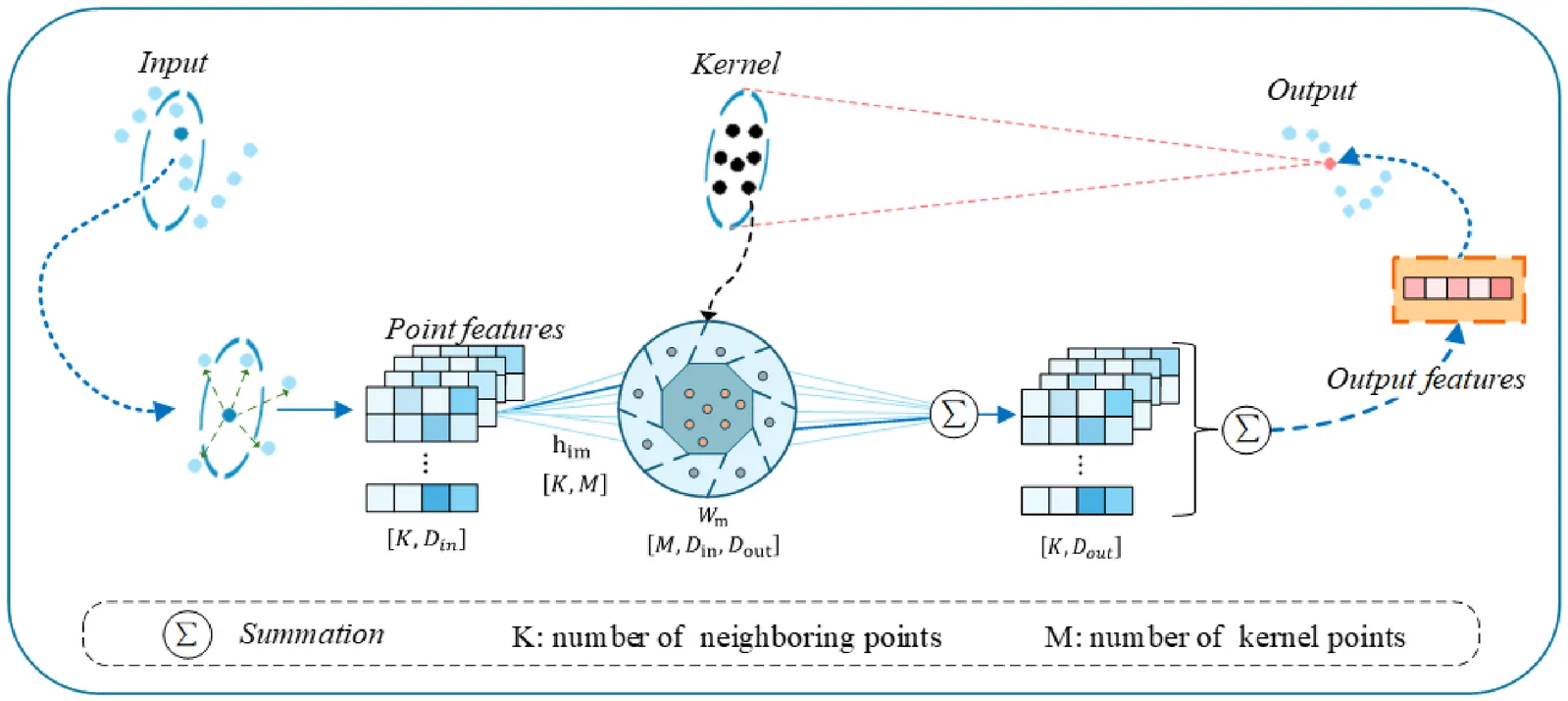
Structure of the KPConv module.

Kernel Point Convolution (KPConv) places a set of virtual kernel points in a local Euclidean space and associates convolution weights with spatial positions. This design allows neighboring points to participate in feature aggregation according to their relative positions with respect to the kernel points. In the improved network, we retain the basic convolution formulation of KPConv and place the module after the first set abstraction layer (SA1) of PointNet++ to strengthen shallow local geometric feature extraction. Compared with the shared MLP and max-pooling operations used directly in the original SA layer, the KPConv module better characterizes local spatial distributions and thus improves the network’s ability to capture hole-wall surface variations, burr protrusion directions, and geometric boundary details. Figure 5 illustrates the structure of the KPConv module.

The KPConv module uses kernel-point correlations to aggregate neighboring point features. Let *x_c_* denote the center point of a local spatial neighborhood. Its neighborhood *N*(*x_c_*) is defined as follows:

(1) $$N_{\left(x_{c}\right)}=\{x_{i}|{∥x_{i}-x_{c}∥}_{2}\leq r\}$$

Here, *x_i_* denotes a neighboring point of center point *x_c_*, *r* denotes the neighborhood search radius, and |⋅|_2_ denotes the Euclidean norm.

In KPConv, M virtual kernel points are set within the local reference coordinate system of the center point *x_c_*, denoted as ${\left\{p_{m}\right\}}_{m=1}^{M}$. Among them, *p_m_* represents the *m*-th virtual kernel point, and M represents the number of kernel points. Each virtual kernel point corresponds to a learnable weight matrix *W_m_*, where $W_{m}\in R^{D_{\mathrm{out}}\times D_{\mathrm{in}}}$, *D_in_* and *D_out_* denote the input and output feature dimensions, respectively. For the neighboring point *x_i_*, its input feature is denoted as *f_i_*, where $f_{i}\in R^{D_{\mathrm{in}}}$.

We first calculate the spatial correlation weight *h_im_* between neighboring point *x_i_* and the *m*-th kernel point as follows:

(2) $$h_{\mathrm{im}}=\max\left(0,1-\frac{{\left\|\left(x_{i}-x_{c}\right)-p_{m}\right\|}_{2}}{\sigma }\right)$$

Here, *x_i_* − *x_c_* denotes the local coordinates of neighboring point *x_i_* relative to center point *x_c_*, *p_m_* denotes the coordinates of the *m*-th kernel point in the local reference system, and *σ* denotes the decay parameter that controls the influence radius of the kernel point.

We then use *h_im_* to weight the neighboring features and apply the corresponding weight matrix *W_m_* for feature transformation. The output feature of center point *x_c_* is expressed as follows:

(3) $$F_{\mathrm{out}}\left(x_{c}\right)=\sum_{x_{i}\in N\left(x_{c}\right)}\sum_{m=1}^{M}h_{\mathrm{im}}W_{m}f_{i}$$

Here, $F_{\mathrm{out}}\left(x_{c}\right)\in R^{D_{\mathrm{out}}}$ denotes the output feature of center point *x_c_*, *h_im_* denotes the correlation weight between neighboring point *x_i_* and the *m*-th virtual kernel point, *f_i_* denotes the input feature of *x_i_*, and *W_m_* denotes the learnable weight matrix associated with the *m*-th virtual kernel point.

For the parameter settings, we set the neighborhood search radius of the KPConv module to 0.1 and the maximum number of neighboring points to 32. Based on the sensitivity analysis of the number of kernel points, we selected 15 kernel points and set the kernel initialization radius to 0.1. During initialization, we placed the first kernel point at the origin of the local coordinate system. We generated the remaining 14 kernel points from random vectors sampled from a three-dimensional Gaussian distribution and normalized them onto the surface of a sphere with a radius of 0.1. This initialization allows the kernel points to cover both the central position and different spatial directions. During training, we treated the kernel-point positions as learnable parameters and updated them through backpropagation, enabling them to adapt their spatial distribution to local geometric features.

#### 3.3.2. KGPA Module

Existing attention mechanisms differ in how they generate attention weights and incorporate geometric information. Squeeze-and-Excitation (SE) [24] and the Convolutional Block Attention Module (CBAM) [25] mainly generate attention weights from existing features. Consequently, burr-point features may not be fully preserved during neighborhood aggregation, making it difficult to accurately identify burr edges and small protrusions. Point Transformer Attention [26] uses the relative positions between center points and neighboring points to strengthen the representation of local spatial structures and point relationships. However, it mainly considers relative positions when calculating attention weights and does not account for variations in local point cloud density. Therefore, existing attention mechanisms do not fully capture the local geometric relationships and density variations in burr point clouds, limiting their ability to highlight critical features such as burr edges and small protrusions.

**Figure 6:**
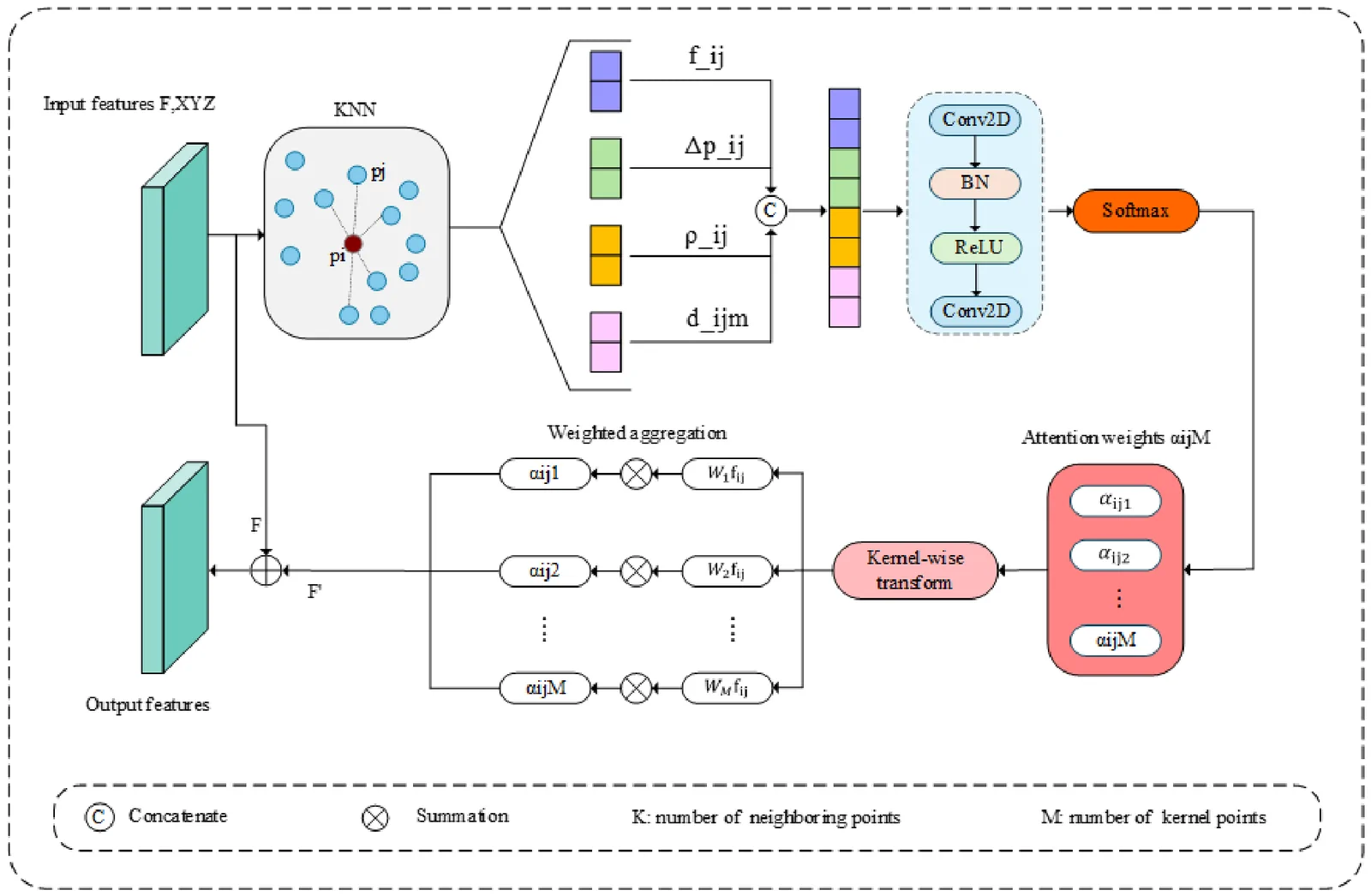
Structure of the KGPA module.

To address these limitations, we developed a Kernel-Guided Geometric Point Attention (KGPA) module inspired by the virtual kernel-point modeling strategy of KPConv. The KGPA module uses virtual kernel points distributed in a local Euclidean space as geometric references and calculates the distances from neighboring points to different kernel points. It then combines point features, relative coordinates, local density, and distances to kernel points to generate attention weights. Compared with methods that calculate attention weights mainly from feature information or center–neighbor relationships, KGPA uses multiple virtual kernel points as local geometric references, jointly incorporates distances to kernel points, relative coordinates, local density, and point features to generate attention weights, and fuses the local responses associated with different kernel points through kernel-wise feature aggregation. This design strengthens the representation of burr edges, small protrusions, and regions with local geometric variations while reducing interference from normal hole-wall points. Figure 6 illustrates the structure of the KGPA module.

The KGPA module uses the k-nearest neighbors (KNN) algorithm to identify neighboring points for each center point and construct a local spatial neighborhood. Let (*K*) denote the number of neighboring points. The relative coordinate between center point (*p_i_*) and its (j)-th neighboring point (*p_j_*) is calculated as follows:

∆*p_ij_* = *p_j_* − *p_i_* (4)

Here, Δ*p_ij_* denotes the three-dimensional vector from center point *p_i_* to neighboring point *p_j_*. This vector provides basic topological information for the subsequent evaluation of local density and calculation of spatial attention distributions.

Because point clouds acquired through actual scanning often have nonuniform local spatial distributions, the KGPA module evaluates local geometric density to prevent high-density regions from dominating feature extraction. It calculates the reciprocal of the squared distance between each neighboring point and the center point and then normalizes the result to obtain the local density weight:

(5) $$\rho_{\mathrm{ij}}=\frac{{\left({\left\|∆p_{\mathrm{ij}}\right\|}_{2}^{2}+\epsilon \right)}^{-1}}{\sum_{k=1}^{K}{\left({\left\|∆p_{\mathrm{ik}}\right\|}_{2}^{2}+\epsilon \right)}^{-1}}$$

Here, ${\left\|\Delta p_{\mathrm{ij}}\right\|}_{2}^{2}$ denotes the squared Euclidean distance between the two points, and *ϵ* denotes a small constant that prevents division by zero. This weight adaptively adjusts the response strength of regions with different point densities during feature aggregation.

To capture spatial geometric distributions along different directions, the KGPA module initializes *M* learnable kernel points in the local space. It calculates the squared Euclidean distance *d_ijm_* between local relative coordinate ∆*p_ij_* and the *m*-th kernel point:

(6) $$d_{\mathrm{ijm}}={\left\|∆p_{\mathrm{ij}}-k_{m}\right\|}_{2}^{2}$$

The KGPA module then concatenates local input feature *f_ij_*, relative coordinate Δ*p_ij_*, density weight *ρ_ij_*, and kernel-point distance *d_ijm_*. It feeds the resulting joint feature into a shared multilayer perceptron implemented using two-dimensional convolutional layers (Conv2D), batch normalization (BN), and a rectified linear unit (ReLU). A Softmax function then generates the multi-kernel attention weight:

*α_ijm_* = *Softmax*(*MLP*(*Concat*(*f_ij_*, ∆*p_ij_*, *ρ_ij_*, *d_ijm_*))) (7)

Here, the Softmax operation normalizes the attention weights across the *M* kernel points.

Finally, the KGPA module uses the learnable weight matrix *W_m_* associated with the *m*-th kernel point to linearly transform local feature *f_ij_*. It then combines the transformed features with attention weight *α_ijm_* to perform weighted aggregation. To maintain stable feature propagation, the module adds the aggregated features to the original input feature *F* through a pointwise residual connection, producing the enhanced local geometric feature:

(8) $$F_{\mathrm{out}}=F+\sum_{j=1}^{K}\sum_{m=1}^{M}\left(\alpha_{\mathrm{ijm}}\cdot \left(W_{m}f_{\mathrm{ij}}\right)\right)$$

For the KGPA module, we set the number of neighboring points (K) for each center point to 16 and the number of kernel points (M) in the local space to 15. The input feature dimension was 128. The attention-weight prediction network consisted of two shared convolutional layers. The first layer had 64 output channels and was followed by batch normalization and ReLU activation. The second layer had 15 output channels, each corresponding to the attention response of one kernel point. Finally, the Softmax function normalized the attention weights.

### 3.4. Evaluation Metrics

To comprehensively evaluate the proposed model, we assessed both segmentation accuracy and computational performance. We used point cloud semantic segmentation metrics to evaluate segmentation accuracy. We also evaluated computational cost and inference efficiency using the number of model parameters, floating-point operations, single-sample inference time, and processing speed. These metrics allowed us to determine whether the inference time would interfere with the actual point cloud acquisition and inspection workflow.

#### 3.4.1. Model Accuracy Evaluation Indicators

We evaluated segmentation accuracy using four metrics: mean intersection over union (mIoU), overall accuracy (OA), precision, and recall. We selected mIoU as the primary evaluation metric because it comprehensively reflects the model’s segmentation performance for both burr and background points. OA measures the proportion of correctly classified points among all points. Precision measures the proportion of correctly classified burr points among all points predicted as burrs and therefore reflects the accuracy of positive predictions. Recall measures the proportion of correctly detected burr points among all ground-truth burr points. We calculated these metrics as follows:

(9) $$\mathrm{mIoU}=\frac{1}{C}\sum_{i=1}^{c}\frac{TP_{i}}{TP_{i}+FP_{i}+FN_{i}}$$

(10) $$\mathrm{OA}=\frac{\mathrm{TP}+\mathrm{TN}}{\mathrm{TP}+\mathrm{TN}+\mathrm{FP}+\mathrm{FN}}$$

(11) $$\mathrm{Precision}=\frac{\mathrm{TP}}{\mathrm{TP}+\mathrm{FP}}$$

(12) $$\mathrm{Recall}=\frac{\mathrm{TP}}{\mathrm{TP}+\mathrm{FN}}$$

Here, *C* denotes the number of classes. In the mIoU calculation, *TP_i_* denotes the number of points correctly classified as class *i*, *FP_i_* denotes the number of points from other classes incorrectly classified as class *i*, and *FN_i_* denotes the number of points from class *i* incorrectly classified as another class. For OA, precision, and recall, burr points are treated as the positive class and background points as the negative class. Accordingly, *TP* denotes the number of burr points correctly predicted as burrs; *FP* denotes the number of background points incorrectly predicted as burrs; *TN* denotes the number of background points correctly predicted as background; and *FN* denotes the number of burr points incorrectly predicted as background.

#### 3.4.2. Model Complexity and Inference Efficiency Metrics

In industrial inspection, a model must achieve high point cloud segmentation accuracy while ensuring that its inference time does not interfere with the point cloud acquisition and inspection workflow. We therefore used the number of model parameters (Params), floating-point operations (FLOPs), single-sample inference time (latency), and processing rate in samples per second (FPS) to evaluate computational complexity and inference efficiency.

Params measures the total number of trainable parameters in the model and reflects model size and storage requirements. We calculated Params as follows:

(13) $$\mathrm{Params}=\sum_{l=1}^{L}P_{l}$$

Here, *L* denotes the total number of layers in the model, and *P_l_* denotes the number of trainable parameters in the *l*-th layer.

FLOPs measure the number of floating-point operations required for one forward inference pass and reflect the computational cost of the model. We calculated FLOPs as follows:

(14) $$\mathrm{FLOPs}=\sum_{l=1}^{L}{\mathrm{FLOPs}}_{l}$$

Here, FLOPs*_l_* denotes the number of floating-point operations required for the forward computation of the *l*-th layer. For ease of comparison, we expressed model complexity in GFLOPs, calculated as follows:

(15) $$\mathrm{GFLOPs}=\frac{\mathrm{FLOPs}}{10^{9}}$$

Latency measures the average time required for the model to process one oil injection-hole point cloud sample and produce its segmentation result. We calculated single-sample latency in milliseconds as follows:

(16) $${\mathrm{Latency}}_{\mathrm{ms}}=\frac{T_{\mathrm{total}}}{N}$$

Here, *T*_total_ denotes the total time required to perform inference on all test samples, in milliseconds; *N* denotes the total number of test samples; and Latency_ms_ denotes the average inference time per point cloud sample.

FPS denotes the number of point cloud samples processed by the model per second and measures inference speed. We calculated FPS as follows:

(17) $$\mathrm{FPS}=\frac{1000}{{\mathrm{Latency}}_{\mathrm{ms}}}$$

### 3.5. Loss Function

We used the standard cross-entropy loss to optimize point-level classification and achieve accurate burr point cloud segmentation:

(18) $$L_{\mathrm{ce}}=-\frac{1}{N}\sum_{i=1}^{N}\sum_{c=1}^{C}y_{\mathrm{ic}}\log\left({\hat{y}}_{\mathrm{ic}}\right)$$

Here, *N* denotes the number of input points, *C* denotes the number of segmentation classes, *y_ic_* denotes the ground-truth indicator for whether the *i*-th point belongs to class *c*, and ${\hat{y}}_{\mathrm{ic}}$ denotes the predicted probability that the *i*-th point belongs to class *c*.

The complex local structures, irregular boundaries, and noise in oil injection-hole burr point clouds may cause the model to overfit local noise or sample-specific features during training. To reduce overfitting and improve training stability, we used the AdamW optimizer with decoupled weight decay. For a compact conceptual representation, the regularized training objective can be approximated as follows:

(19) $$L_{\mathrm{total}}=L_{\mathrm{ce}}+\lambda \sum_{l=1}^{L}{\left\|W_{l}\right\|}_{2}^{2}$$

Here, *L*_total_ denotes the approximate total training objective, *L*_ce_ denotes the cross-entropy loss, *L* denotes the number of layers subject to weight decay, *W_l_* denotes the weight parameters of the *l*-th layer, and *λ* denotes the weight-decay coefficient.

## 4. Results

### 4.1. Dataset Split and Experimental Settings

#### 4.1.1. Dataset Split

The oil injection-hole burr point cloud dataset contained 1040 samples representing diverse burr morphologies, varying extents of burr distribution along the hole edge, and different degrees of local protrusion. The dataset therefore covered a range of typical machining-induced burr morphologies. For the comparative and ablation experiments, we randomly divided the dataset in an 8:2 ratio into a training set containing 832 samples and a test set containing 208 samples. The dataset was partitioned at the individual point-cloud sample level. Each sample was assigned exclusively to one subset, and no identical point-cloud sample was shared between the training and test sets. The partitioning was completed before online data augmentation. Because the dataset was split at the individual point-cloud sample level, different samples from the same wheelset or oil-injection hole could have been assigned to the training and test sets. Accordingly, this evaluation measures performance on held-out point-cloud samples from the available dataset and does not establish generalization to entirely unseen wheelsets or oil-injection holes. We used a fixed random seed of 42 for all experiments to ensure consistency and reproducibility. All models used the same data split and number of input points. All models were trained and evaluated on the same hardware platform. Their training settings and relevant model-specific parameters are summarized in Table 3.

During training, we applied online data augmentation only to the training samples. Specifically, we randomly scaled each input point cloud using a scale factor sampled from [0.9, 1.1], randomly rotated it about the Z-axis by an angle sampled from [0, 2*π*], and randomly translated it along each of the three coordinate axes within [−0.1, 0.1]. We also added Gaussian noise with a standard deviation of 0.01 to each point coordinate and clipped the noise values to [−0.05, 0.05]. These operations increased the variations in point cloud scale, orientation, position, and local perturbations. Under the hardware and parameter settings described above, training the proposed model for 100 epochs required approximately 2 h, corresponding to an average of approximately 72 s per epoch.

#### 4.1.2. Experimental Parameter Settings

We implemented all network architectures using the PyTorch deep learning framework and trained and evaluated them on a workstation equipped with an NVIDIA GeForce RTX 4060 GPU and an Intel Core i9-13980HX processor. We optimized the proposed network using AdamW with a weight-decay coefficient of 1 × 10^−4^ and coefficients *β*_1_ and *β*_2_ set to 0.9 and 0.999, respectively. We set the initial learning rate to 0.001 and used a cosine annealing schedule to dynamically adjust it during training, with a minimum learning rate of 0.0001. To ensure stable convergence, we used a batch size of 16 and trained the network for 100 epochs. Table 3 summarizes the main training settings of the comparison models and the proposed method, including the optimizer, learning-rate schedule, batch size, number of epochs, weight decay, and key model-specific parameters. All models used the same dataset split, number of input points, and evaluation protocol.

### 4.2. Comparative Experiments

#### 4.2.1. Performance Comparison of Point Cloud Segmentation Networks

We evaluated PointNet [27], RandLA-Net [28], PointNeXt [29], Point Transformer [26], DeepLA-Net [30], PointNet++ [9], and DGCNN [31] on the oil injection-hole burr dataset. Table 4 presents the segmentation results.

The models exhibited clear differences in segmentation performance. PointNet achieved an mIoU of only 81.65%, indicating that global feature aggregation alone cannot adequately represent the local geometric variations in burr regions. PointNeXt, Point Transformer, and RandLA-Net outperformed PointNet, but their overall segmentation accuracy remained lower than that of PointNet++, DeepLA-Net, and DGCNN. PointNet++, DeepLA-Net, and DGCNN achieved higher segmentation accuracy because their hierarchical feature extraction, local neighborhood modeling, or multiscale feature aggregation enabled them to extract local point cloud structures more effectively. Among the comparison models, DGCNN achieved the highest mIoU of 92.55%, which was still 1.70 percentage points lower than that of the proposed model. These results indicate that introducing the KPConv and KGPA modules into PointNet++ further strengthens local geometric feature extraction and the representation of critical burr regions, thereby improving the segmentation of oil injection-hole burrs on the evaluated dataset.

**Figure 7:**
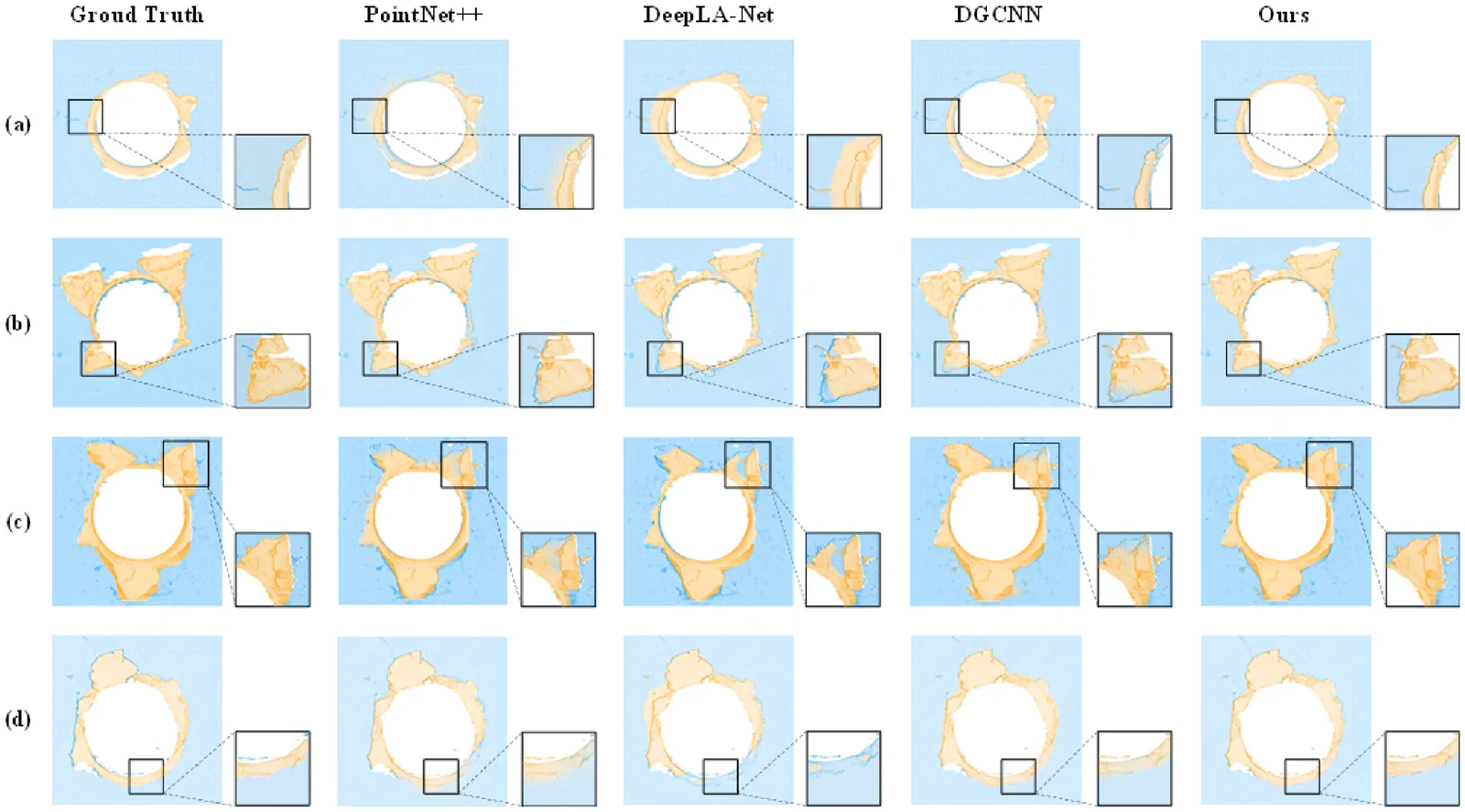
Comparison of segmentation results produced by different models for four oil injection-hole burr samples: (**a**) sample 1; (**b**) sample 2; (**c**) sample 3; and (**d**) sample 4. Yellow indicates burr points, and blue indicates hole-wall points.

The visualization results in Figure 7 show that PointNet++ identified the main burr regions but produced incomplete boundaries and local misclassifications. DeepLA-Net produced better overall segmentation results but could not fully distinguish burr points from normal hole-wall points in several small regions, as shown in Figure 7c,d. Although DGCNN achieved an mIoU of 92.55%, it still produced local misclassifications around some burr boundaries, as shown in Figure 7a. By contrast, the predictions of the proposed model were closer to the ground-truth labels across burr samples with different morphologies and preserved the burr regions more completely.

#### 4.2.2. Comparison of Attention Mechanisms

To further evaluate the specific contribution of the KGPA module to oil injection-hole burr segmentation, we compared it with representative attention mechanisms, including SE, CBAM, and Point Transformer Attention. We used the model containing the KPConv module as the baseline and placed each attention module after FP1. We kept the remaining network architecture and experimental settings unchanged and replaced only the attention module. Table 5 presents the results.

All attention mechanisms improved the segmentation performance of the baseline model. SE and CBAM achieved mIoU values of 93.16% and 93.45%, respectively, whereas Point Transformer Attention further increased the mIoU to 94.13%. The KGPA module achieved the highest mIoU, OA, and recall, reaching 94.25%, 98.38%, and 98.65%, respectively. Its precision was 98.50%, which was comparable to the 98.52% achieved by Point Transformer Attention. By incorporating distances to kernel points and local density information, the KGPA module further strengthened the representation of local burr geometry and achieved the best overall performance on the oil injection-hole burr point cloud segmentation task.

### 4.3. Ablation Experiments

#### 4.3.1. Ablation Study of the Core Modules

To evaluate the contributions of the proposed modules, we conducted ablation experiments using PointNet++ as the baseline. We added the KPConv and KGPA modules individually and in combination to assess their effects on oil injection-hole burr point cloud segmentation. Table 6 presents the results.

After introducing only the KPConv module, the model achieved an mIoU of 92.76%, an OA of 97.28%, a recall of 97.05%, and a precision of 97.12%. These values exceeded those of the baseline by 0.79, 0.52, 0.65, and 0.96 percentage points, respectively, indicating that the KPConv module strengthens local geometric feature extraction. After introducing only the KGPA module, the four metrics reached 93.38%, 97.85%, 97.92%, and 97.80%, representing improvements of 1.41, 1.09, 1.52, and 1.64 percentage points, respectively, over the baseline. These results indicate that KGPA adaptively weights neighboring-point features, thereby strengthening feature representation in critical regions such as burr boundaries and small protrusions. When we incorporated both modules, the model achieved the highest mIoU, OA, recall, and precision of 94.25%, 98.38%, 98.65%, and 98.50%, respectively. These results demonstrate that KPConv’s extraction of local geometric features complements KGPA’s enhancement of critical-region features and that combining the two modules further improves burr point cloud segmentation performance.

#### 4.3.2. Internal Structure Ablation of KGPA

The KGPA module consists of four parts: kernel distance feature, relative coordinate feature, density feature, and kernel-wise feature aggregation. In order to evaluate the contribution of each component to improving the performance of the model, ablation experiments were conducted. The results are shown in Table 7.

Removing any component from the KGPA module reduced the mIoU, recall, and precision to varying degrees, indicating that all four components contributed positively to model performance. Removing the kernel-distance feature reduced the mIoU from 94.25% to 92.39%, a decrease of 1.86 percentage points and the largest reduction among the four variants. This result indicates that distances to kernel points provide local geometric references for neighboring points and play an important role in representing features of burr regions. Removing the relative-coordinate feature, density feature, and kernel-wise feature aggregation reduced the mIoU to 93.94%, 93.89%, and 93.91%, respectively, showing that these components also improve local feature representation. The full KGPA module achieved the highest mIoU, recall, and precision, indicating that the joint use of all four components strengthens feature representation in critical burr regions and thereby improves segmentation performance.

### 4.4. Sensitivity Analysis of KPConv Kernel Number

The local feature extraction performance of the KPConv module depends on the number of kernel points M, the search radius r, and the maximum number of neighboring points K. The number of kernel points determines the level of detail with which the module represents local geometric structures, the search radius determines the spatial extent of each neighborhood, and the maximum number of neighboring points determines how many points participate in local feature aggregation. To evaluate the effect of M on model performance, we varied M while fixing r at 0.1 and K at 32. Table 8 presents the results.

### 4.5. Five-Fold Cross-Validation and Significance Analysis

#### 4.5.1. Cross-Validation Results

To further evaluate the stability of the proposed model across different dataset partitions and reduce the influence of a single random dataset partition, we conducted five-fold cross-validation on the burr point cloud dataset. The dataset was partitioned at the individual point-cloud sample level into five non-overlapping subsets, with each sample assigned to only one subset.

The five-fold cross-validation results are presented in Table 9. The proposed model maintained high OA, precision, recall, and mIoU values across all five folds. Its mIoU ranged from 94.08% to 94.25%, corresponding to a range of only 0.17 percentage points. Moreover, it achieved a higher mIoU than PointNet++, DGCNN, and Point Transformer in every fold, indicating that its performance advantage was consistent across the different dataset partitions.

To compare the overall cross-validation performance of the models, we calculated the mean and sample standard deviation of each metric across the five folds, as summarized in Table 10.

The proposed model achieved mean mIoU, OA, precision, and recall values of 94.16%, 98.30%, 98.42%, and 98.54%, respectively, outperforming PointNet++, DGCNN, and Point Transformer on all four metrics. The sample standard deviation of its mIoU was only 0.07 percentage points, indicating limited variation across the different dataset partitions.

#### 4.5.2. Paired t-Test Results

Although the five-fold cross-validation results demonstrated the stability of the proposed model, we further examined whether its performance differences relative to the comparison models were statistically significant. We conducted two-sided paired t-tests between the proposed model and PointNet++, DGCNN, and Point Transformer. Table 11 presents the results.

All paired comparisons showed statistically significant differences *p* < 0.001. For mIoU, the proposed model outperformed PointNet++, DGCNN, and Point Transformer by mean values of 2.33, 1.85, and 5.85 percentage points, respectively. These results indicate that the proposed method maintained its segmentation advantage across the evaluated sample-level partitions of this dataset.

### 4.6. Analysis of Model Complexity and Inference Efficiency

For oil injection-hole burr point cloud segmentation, a model must balance segmentation accuracy with computational complexity and inference efficiency. To quantify the computational cost introduced by the proposed modules, we compared different models in terms of the number of parameters, floating-point operations, single-sample inference latency, and the number of samples processed per second. We conducted all experiments using the same hardware platform and test data. We set every model to evaluation mode and disabled gradient computation during inference. Table 12 presents the results.

The models differed substantially in computational complexity and inference efficiency. RandLA-Net had the fewest parameters and the lowest computational cost, with 0.04 million parameters and 0.14 GFLOPs, respectively. PointNet achieved the shortest single-sample inference latency of 5.27 ms. Compared with PointNet++, the proposed model increased the number of parameters from 1.73 million to 2.37 million, GFLOPs from 9.77 to 10.81, and single-sample inference latency from 174.28 ms to 268.00 ms. Although the KPConv and KGPA modules increased computational cost and inference time, they improved the mIoU from 91.97% to 94.25%, an increase of 2.28 percentage points. Overall, the proposed modules introduce additional computational overhead while improving burr segmentation, demonstrating a trade-off between segmentation accuracy and computational cost.

### 4.7. Model Limitations

**Figure 8:**
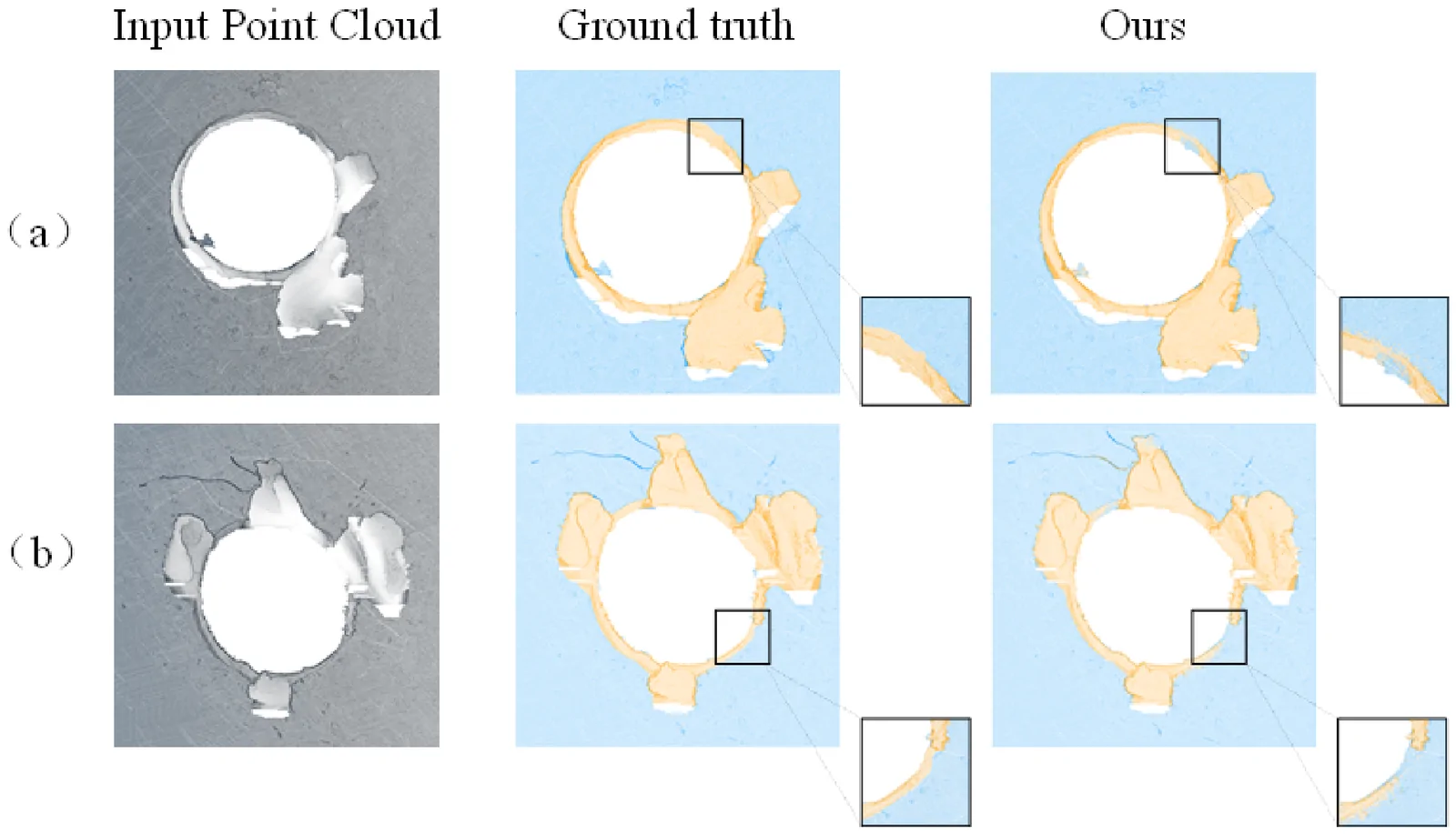
Segmentation results for challenging samples: (**a**) sample with small burrs; (**b**) sample with irregular burr boundaries.

We visually analyzed challenging samples containing extremely small burrs and irregular boundaries, as shown in Figure 8. In Figure 8a, the burr region is small and directly connected to the normal hole wall. Although the overall prediction closely matches the ground-truth annotation, the enlarged view reveals that the model fails to identify a small number of boundary regions accurately. In Figure 8b, the burr has an irregular boundary, and the local prediction differs from the ground-truth annotation, with several boundary regions remaining inaccurately segmented.

These results show that the proposed method generally segments burrs effectively but still produces errors for extremely small burrs and irregular boundary regions. Therefore, its fine-grained segmentation capability requires further improvement.

## 5. Discussion

### 5.1. Analysis of Oil Injection-Hole Burr Segmentation Performance

Oil injection-hole burrs are typically small, irregularly shaped, and directly connected to the normal hole wall. The subtle local geometric differences between burrs and the hole wall make point cloud segmentation challenging. The proposed method achieved an mIoU of 94.25%, an OA of 98.38%, a recall of 98.65%, and a precision of 98.50%. Its mIoU exceeded those of PointNet++ and DGCNN by 2.28 and 1.70 percentage points, respectively, indicating that the improved network distinguishes burr regions from the normal hole wall more effectively.

Although PointNet++ extracts basic local features from oil injection-hole point clouds, it still produces misclassifications and missed detections around burr boundaries, small protrusions, and regions with subtle local geometric variations. We introduced the KPConv module during shallow feature extraction and used the KGPA module to enhance critical regional features, enabling the network to exploit local geometric information around burrs more effectively. Consequently, the proposed method produced better segmentation results for both overall burr regions and local boundaries. These findings indicate that strengthening local geometric structure representation and critical-region features improves the segmentation of small local defects such as oil injection-hole burrs.

### 5.2. Roles of the KPConv and KGPA Modules

The ablation results show that both the KPConv and KGPA modules improve oil injection-hole burr segmentation, although they play different roles. The KPConv module aggregates local features according to the spatial relationships between virtual kernel points and neighboring points. This process allows the network to characterize local structures such as burr protrusions, hole-wall curvature variations, and boundary transitions more effectively. Introducing only the KPConv module increased the mIoU from 91.97% to 92.76%, indicating that stronger local geometric feature extraction improves burr recognition.

By contrast, the KGPA module focuses on differences in the contributions of individual neighboring points to burr segmentation. In addition to point features and relative coordinates, it incorporates local density and distances to kernel points. It then uses kernel-wise feature aggregation to calculate neighboring-point weights, allowing attention allocation to reflect both spatial relationships and local distribution variations. In the attention-mechanism comparison, the KGPA module achieved the highest mIoU, OA, and recall, indicating that the joint use of these geometric cues better captures the local structural characteristics of oil injection-hole burrs.

The internal ablation results further demonstrate the contributions of different geometric cues. Removing the kernel-distance feature reduced the mIoU from 94.25% to 92.39%, the largest decrease among the tested components, indicating that kernel points provide effective local geometric references for neighboring points. Removing relative coordinates, density features, or kernel-wise feature aggregation also reduced model performance to varying degrees. These results show that a single type of spatial information cannot fully characterize the subtle differences between burrs and the hole wall. Combining multiple local geometric cues during feature weighting improves the recognition of burr boundaries and small protrusions.

### 5.3. Stability Across Different Dataset Partitions

The five-fold cross-validation results show that the proposed method maintained stable segmentation performance across different dataset partitions. Its mIoU ranged from 94.08% to 94.25%, with a mean of 94.16% and a standard deviation of only 0.07. The mean mIoU exceeded those of PointNet++, DGCNN, and Point Transformer by 2.33, 1.85, and 5.85 percentage points, respectively. Moreover, the paired comparisons yielded *p* < 0.001 across the evaluated sample-level folds, supporting a consistent performance difference within this dataset.

### 5.4. Analysis of Model Calculation Complexity and Application Limitations

The KPConv and KGPA modules require more extensive extraction and aggregation of local geometric information from burr regions and therefore introduce additional computational overhead. Compared with PointNet++, the proposed model increases the number of parameters from 1.73 M to 2.37 M, the computational cost from 9.77 to 10.81 GFLOPs, and the single-sample inference latency from 174.28 to 268.00 ms. Meanwhile, the mIoU increases from 91.97% to 94.25%, corresponding to an improvement of 2.28 percentage points. These results demonstrate a trade-off between segmentation accuracy and computational cost.

To further examine the influence of the inference latency on the inspection workflow, we estimated the duration of the principal stages using the current experimental procedure. The robot requires approximately 5 s to move the laser sensor to the predefined acquisition position, while scanning one oil-injection-hole burr region requires approximately 2–3 s. The proposed model requires 0.268 s to process one point cloud sample. Assuming that these stages are executed sequentially, the estimated duration of the principal inspection stages is therefore approximately 7.27–8.27 s per sample. Model inference accounts for approximately 3.2–3.7% of this duration, indicating that it is not the dominant time-consuming stage in the current experimental workflow.

This estimate is based on the present experimental platform rather than a fully deployed production line. Auxiliary operations, such as data transmission and point cloud preprocessing, were not timed separately. Therefore, whether the proposed method satisfies the production-cycle requirements of a specific industrial site must be further evaluated under the corresponding equipment configuration, robot trajectory, scanning parameters, and operating conditions. Future work will focus on end-to-end timing and optimization of the complete inspection system.

### 5.5. Model Limitations and Future Work

The proposed model still has certain limitations when processing extremely small burrs and samples with irregular boundaries. For small burrs, the model may fail to segment some local regions. When burr boundaries are highly irregular, the predicted boundaries may still deviate from the ground-truth annotations. These results indicate that, although the proposed method can effectively identify the main burr regions, further improvements are required in preserving boundary completeness for extremely small burrs and achieving fine-grained segmentation of irregular boundaries.

In addition, the dataset is relatively small for deep learning-based point cloud segmentation. Although the existing samples include diverse burr morphologies, varying extents of burr distribution along the hole edge, and different degrees of local protrusion, they may not fully cover all burr morphologies encountered in actual machining processes. Therefore, the current experimental results remain limited by the dataset size and sample diversity. The sample-level split also did not separately hold out complete wheelsets or oil-injection holes. Consequently, the present results cannot determine how well the model would perform on entirely unseen wheelsets or oil-injection holes.

Furthermore, all experimental data were acquired using the same line laser sensor and acquisition platform, and the performance of the proposed model under different scanning devices and acquisition conditions has not yet been validated. Therefore, the current experimental results mainly reflect the model’s performance within the scope of the existing acquisition platform and dataset. Future work will acquire point cloud data using different scanning devices and under a wider range of acquisition conditions, expand the dataset size and sample diversity, and further improve the model’s ability to segment extremely small burrs and irregular boundaries, thereby enhancing its applicability to practical industrial inspection.

## 6. Conclusions

Accurate segmentation of oil injection-hole burrs provides an essential foundation for automated burr inspection, localization, and subsequent processing. To address the small size, irregular morphology, and direct connection of burrs to the normal hole wall, we proposed an improved PointNet++-based method for segmenting oil injection-hole burr point clouds.

We introduced a KPConv module into the shallow feature extraction stage of PointNet++ to strengthen local geometric feature extraction. We also designed a KGPA module that integrates relative coordinates, local density, and distances to kernel points, thereby improving the network’s ability to identify burr boundaries and small protrusions. The proposed method achieved an mIoU of 94.25%, an OA of 98.38%, a recall of 98.65%, and a precision of 98.50%. Its mIoU exceeded that of PointNet++ by 2.28 percentage points. The five-fold cross-validation results showed consistent performance across different sample-level partitions of the available dataset.

Overall, the proposed method improves the accuracy of oil injection-hole burr point cloud segmentation and provides accurate point cloud information for subsequent burr localization and processing. However, the model still produces local segmentation errors for extremely small burrs and irregular boundaries. Its performance on entirely unseen wheelsets or oil-injection holes, and with different scanning devices, has not yet been evaluated. Future work will incorporate data acquired using different devices and under more diverse acquisition conditions and will further improve the model’s ability to identify complex burr regions.

## Figures and Tables

**Table 1:** SR7020 Line Laser Sensor Device Parameters.

Parameter	Measurement Conditions	Value
Model	—	SR7020
Reference distance	—	23 mm
Measurement range	*Z*-axis (height), near side	3.8 mm
	*Z*-axis (height), far side	9.2 mm
Measurement width	*X*-axis, at reference distance	9.6 mm
	*X*-axis, far side	9.6 mm
Repeatability	*Z*-axis (height)	0.1 μm
	*X*-axis (width)	1.0 μm
Linearity	*Z*-axis (height)	±0.05% F.S
Profile data interval	*X*-axis (width)	3 μm
Number of profile points	*X*-axis	3200
Profile frequency	—	750–2000 Hz

**Table 2:** Statistical Table of Burr Depth Information.

Statistic	Value (mm)
Minimum	0.410
Maximum	1.099
Mean	0.816
Standard deviation	0.117

**Table 3:** Training settings of the proposed method and comparison models.

Model	Optimizer (LR)	Batch	Epochs	WD	Key Model Settings
PointNet	Adam (0.001)	16	100	1 × 10^−4^	Global dim. 512; dropout 0.4
RandLA-Net	Adam (0.001)	16	100	1 × 10^−3^	KNN k = 16 (deep stage: 8)
PointNeXt	AdamW (0.002)	16	100	1 × 10^−4^	Expansion 4; neighbors 32; radii 0.2/0.4/0.8
Point Transformer	AdamW (0.0005)	16	100	1 × 10^−4^	KNN k = 16; heads 4; dim.64
PointNet++	Adam (0.001)	16	100	1 × 10^−4^	MSG radii: [0.1, 0.2, 0.4]/[0.4, 0.8]; neighbors [32, 64, 128] [64, 128]
DeepLA-Net	AdamW (0.001)	16	100	1 × 10^−4^	8 ResLFE blocks; HDS off
DGCNN	AdamW (0.001)	16	100	1 × 10^−4^	KNN k = 20; embedding dim.1024; dropout 0.5
Ours	AdamW (0.001)	16	100	1 × 10^−4^	KPConv M = 15; KGPA k = 16, M = 15

Note: LR, initial learning rate; WD, weight decay; KNN, k-nearest neighbors; MSG, multi-scale grouping; dim, dimension; ResLFE, residual local feature extraction; HDS, hybrid deep supervision; KPConv, kernel point convolution; KGPA, kernel-guided geometric point attention; k, number of nearest neighbors; M, number of kernel points. All models used a cosine annealing learning-rate schedule.

**Table 4:** Test results of different point cloud segmentation networks on burr dataset.

Method	mIoU	OA	Recall	Precision
PointNet	81.65	90.31	89.65	89.92
RandLA-Net	90.35	95.10	94.86	95.12
PointNeXt	85.34	92.20	91.50	91.85
Point Transformer	88.62	93.68	93.20	93.58
PointNet++	91.97	96.76	96.40	96.16
DeepLA-Net	92.15	97.10	96.75	96.85
DGCNN	92.55	97.52	97.25	97.40
Ours	94.25	98.38	98.65	98.50

**Table 5:** Performance comparison of different attention mechanisms.

Attention Mechanism	mIoU (%)	OA (%)	Recall (%)	Precision (%)
-	92.76	97.28	97.05	97.12
SE	93.16	97.58	97.48	97.49
CBAM	93.45	97.79	97.79	97.75
Point Transformer Attention	94.13	98.27	98.47	98.52
KGPA	94.25	98.38	98.65	98.50

**Table 6:** Ablation results for the core modules.

Method	KPConv	KGPA	mIoU (%)	OA (%)	Recall (%)	Precision (%)
PointNet++	×	×	91.97	96.76	96.40	96.16
Model A	√	×	92.76	97.28	97.05	97.12
Model B	×	√	93.38	97.85	97.92	97.80
Ours	√	√	94.25	98.38	98.65	98.50

Note: √ indicates that the corresponding module is included, while × indicates that it is not included.

**Table 7:** Ablation results for the internal components of the KGPA module.

Variant	KD	RC	DF	KWA	mIoU (%)	Recall (%)	Precision (%)
w/o KD	×	√	√	√	92.39	97.75	96.63
w/o RC	√	×	√	√	93.94	97.36	96.61
w/o DF	√	√	×	√	93.89	97.43	96.67
w/o KWA	√	√	√	×	93.91	97.08	96.88
Full KGPA	√	√	√	√	94.25	98.65	98.50

Note: KD denotes kernel distance, RC denotes relative coordinate, DF denotes density feature, and KWA denotes kernel-wise feature aggregation. √ indicates that the corresponding module is included, while × indicates that it is not included.

**Table 8:** Sensitivity analysis of the number of kernel points in KPConv.

M	r	K	mIoU (%)	Recall (%)	Precision (%)	Params (10^6^)	GFLOPs	Latency (ms)
5	0.1	32	93.80	95.54	93.79	2.209	10.725	220.96
10	0.1	32	93.99	95.43	93.84	2.292	10.767	244.55
15	0.1	32	94.25	98.65	98.50	2.374	10.809	268.00
27	0.1	32	94.08	97.52	97.81	2.573	10.909	342.63

As the number of kernel points increased, the segmentation performance initially improved and then decreased slightly. Increasing M from 5 to 15 raised the mIoU from 93.80% to 94.25%, with recall and precision reaching 98.65% and 98.50%, respectively. These results indicate that an appropriate increase in the number of kernel points improves segmentation performance. When M was further increased to 27, the mIoU decreased to 94.08%, while the number of parameters, GFLOPs, and inference latency all increased without any further improvement in segmentation accuracy. Considering segmentation accuracy, model complexity, and inference latency, we selected M = 15.

**Table 9:** Five-fold Cross-Validation.

Model	Metric	Fold 1	Fold 2	Fold 3	Fold 4	Fold 5
Point Transformer	OA	93.35	93.57	93.18	93.31	93.68
	Precision	93.26	93.44	93.10	93.12	93.58
	Recall	92.91	93.10	92.77	92.82	93.20
	mIoU	88.15	88.63	88.04	88.11	88.62
PointNet++	OA	96.35	96.72	96.58	96.84	96.76
	Precision	95.82	96.23	96.09	96.45	96.16
	Recall	95.89	96.25	96.02	96.49	96.40
	mIoU	91.56	91.85	91.72	92.05	91.97
DGCNN	OA	97.31	97.49	97.12	97.26	97.52
	Precision	97.08	97.36	96.97	97.24	97.40
	Recall	96.91	97.28	96.83	97.01	97.25
	mIoU	92.07	92.48	92.23	92.22	92.55
Ours	OA	98.35	98.31	98.22	98.24	98.38
	Precision	98.42	98.45	98.34	98.39	98.50
	Recall	98.52	98.59	98.46	98.48	98.65
	mIoU	94.15	94.20	94.08	94.10	94.25

**Table 10:** Statistical summary of the five-fold cross-validation results.

Model	mIoU	OA	Precision	Recall
Point Transformer	88.31 ± 0.29	93.42 ± 0.20	93.30 ± 0.21	92.96 ± 0.18
PointNet++	91.83 ± 0.20	96.65 ± 0.19	96.15 ± 0.23	96.21 ± 0.25
DGCNN	92.31 ± 0.20	97.34 ± 0.17	97.21 ± 0.18	97.06 ± 0.20
Ours	94.16 ± 0.07	98.30 ± 0.07	98.42 ± 0.06	98.54 ± 0.08

Note: Values are reported as the mean ± sample standard deviation over the five folds.

**Table 11:** Statistical significance analysis.

Comparison	Metric	Mean Diff	df	t-Value	*p*-Value	Significance
Ours vs. Point Transformer	mIoU	5.85	4	57.75	<0.001	***
Ours vs. DGCNN	mIoU	1.85	4	27.05	<0.001	***
Ours vs. PointNet++	mIoU	2.33	4	26.88	<0.001	***
Ours vs. Point Transformer	OA	4.88	4	71.03	<0.001	***
Ours vs. DGCNN	OA	0.96	4	18.14	<0.001	***
Ours vs. PointNet++	OA	1.65	4	16.95	<0.001	***
Ours vs. Point Transformer	Precision	5.12	4	76.07	<0.001	***
Ours vs. DGCNN	Precision	1.21	4	20.08	<0.001	***
Ours vs. PointNet++	Precision	2.27	4	21.37	<0.001	***
Ours vs. Point Transformer	Recall	5.58	4	118.43	<0.001	***
Ours vs. DGCNN	Recall	1.48	4	24.28	<0.001	***
Ours vs. PointNet++	Recall	2.33	4	22.01	<0.001	***

Note: We used two-sided paired t-tests with df = 4 and a significance level of 0.05. *** indicates *p* < 0.001.

**Table 12:** Computational complexity and inference efficiency of different networks.

Method	Params (M)	GFLOPs	Latency (ms)	FPS
PointNet	0.54	1.53	5.27	189.62
RandLA-Net	0.04	0.14	38.00	26.32
PointNeXt	24.12	4.24	115.71	8.64
DeepLA-Net	0.12	0.48	45.30	22.07
Point Transformer	3.06	2.54	155.47	6.43
PointNet++	1.73	9.77	174.28	5.74
DGCNN	1.80	15.15	195.42	5.12
Ours	2.37	10.81	268.00	3.73

## Data Availability

The original contributions presented in this study are included in the article. Further inquiries can be directed to the corresponding author.
